# Comparative Analysis of the Biochemical Composition, Amino Acid, and Fatty Acid Contents of Diploid, Triploid, and Tetraploid *Crassostrea gigas*

**DOI:** 10.3390/molecules29112671

**Published:** 2024-06-05

**Authors:** Jingjing Fu, Enshuo Zhang, Wensong Yu, Weijun Wang, Youmei Sun, Luyao Dong, Yousen Zhang, Guohua Sun, Zan Li, Qihao Luo, Jianmin Yang

**Affiliations:** 1 School of Agriculture, Ludong University, Yantai 264025, China; fjj000117@163.com (J.F.); zenshuo1998@163.com (E.Z.); sunyoumei4554@163.com (Y.S.); 19909024486@163.com (Y.Z.); sgh_smile@163.com (G.S.); lizanlxm@163.com (Z.L.); 2 Yantai Marine Economic Research Institute, Yantai 264003, China; wgt2000@163.com; 3 College of Fisheries and Life Science, Shanghai Ocean University, Shanghai 201306, China; dly6568@163.com; 4 Yantai Haiyu Marine Technology Co., Ltd., Yantai 264000, China; 17854266371@163.com

**Keywords:** ploidy, *Crassostrea gigas*, amino acid, fatty acid, nutritional quality

## Abstract

Tetraploid oysters are artificially produced oysters that do not exist in nature. The successful breeding of 100% triploid oysters resolved the difficulties of traditional drug-induced triploids, such as the presence of drug residues and a low triploid induction rate. However, little is known concerning the biochemical composition and nutrient contents of such tetraploids. Therefore, we investigated compositional differences among diploid, triploid, and tetraploid *Crassostrea gigas* as well as between males and females of diploids and tetraploids. The findings indicated that glycogen, EPA, ∑PUFA, and omega-3 contents were significantly higher in triploid oysters than in diploids or tetraploids; tetraploid oysters had a significantly higher protein content, C14:0, essential amino acid, and flavor-presenting amino acid contents than diploids or triploids. For both diploid and tetraploids, females had significantly higher levels of glutamate, methionine, and phenylalanine than males but lower levels of glycine and alanine. In addition, female oysters had significantly more EPA, DHA, omega-3, and total fatty acids, a result that may be due to the fact that gonadal development in male oysters requires more energy to sustain growth, consumes greater amounts of nutrients, and accumulates more proteins. With these results, important information is provided on the production of *C. gigas*, as well as on the basis and backing for the genetic breeding of oysters.

## 1. Introduction

Improved standards of living have produced a growing demand for high-quality protein sources and a more balanced diet containing nutrients such as proteins and fatty acids. Therefore, increasing numbers of people have chosen to eat seafood due to its high omega-3 and protein contents. Oysters are considered a delicacy and are often directly eaten raw in order to maintain their fresh and sweet taste. In northern China, *Crassostrea gigas* is a cultivated shellfish of great economic relevance. However, diploid oysters reproduce in the summer, resulting in the discharge of the gonads, making the oyster unsuitable for eating due to poor taste, resulting in a half-year gap in the market. Triploid oysters, in contrast, have been a focus of farmers and researchers due to their sterility, rapid growth, and the consequent ability to maintain a fresh and sweet taste during the reproductive phase. The induction of diploid oysters to triploids using Cytochalasin B (CB) has problems such as drug residues and an unsafe operation process. All-triploid oysters, which were obtained by crossing diploid oysters with tetraploid oysters, rapidly entered commercial aquaculture market [1]. Although researchers from various countries have analyzed the sterility and growth of polyploid oysters [2,3] research regarding the physiology and biochemistry of polyploids is scarce, and research on tetraploid oysters is even rarer. Yue et al. investigated the crossbreeding between tetraploid *C. gigas* and *C. angulata* and described the phenotypic characteristics of their hybrid tetraploids offspring [4]. Li et al. explored the effects of inbreeding on fertilization, growth, and survival in tetraploid *C. gigas* [5]. Our research group studied autotetraploid genotyping technology and obtained the genetic parameters for the growth traits of autotetraploid *C. gigas* (unpublished data). As a crucial germplasm, tetraploid oysters play an important role in breeding triploid oysters to improve the production and quality of oysters. Therefore, further research on the breeding and cultivation of tetraploid oysters is warranted to fully explore their applications in marine aquaculture.

Glycogen is a branched-chain glucose polymer [6] whose storage and utilization are closely related to oyster reproduction [7]. A high glycogen content contributes to the flavor and taste of oysters [8]. Amino acid contents have a significant part in determining the nutritional quality of foods [9] and are the basic units of proteins and peptides [10]. In oysters, protein composition is closely related to gonadal development [11]. The dietary value and health effects of seafood, in particular oysters, are largely related to their content of essential fatty acids (EFAs) and essential amino acids (EAAs). Further, the structures of amino acids determine the flavor of oysters; in particular, the hydrophobicity of amino acid R groups is related to the flavor. According to the level of hydrophobicity, oysters can have a sour or bitter taste. When the R group is an acidic group such as glutamic acid or aspartic acid, oysters have a sour flavor. The free amino acid that has a bitter and sweet taste is arginine [12]. Lipids play an essential part in gamete forming and membrane construction [11]. Polyunsaturated fatty acids (PUFAs) are essential to the growth, ontogeny, and reproduction of living organisms [13] Eicosapentaenoic acid (EPA) and docosahexaenoic acid (DHA) protect against coronary heart disease, cancer, and diabetes [14,15], and a high ratio of ω-3/ω-6 has been demonstrated to contribute to a reduction in the incidence of a number of diseases [16]. However, there are no studies comparing the differences between tetraploid *C. gigas* oysters with other ploidies with respect to their biochemistry or fatty acid composition, and there are no relevant studies reporting differences between tetraploid males and females. Qin et al. (2018) [17] demonstrated that triploid *C. hongkongensis* was superior to diploids regarding protein, fatty acid, and glycogen content. We compared and evaluated the performance of different *C. gigas* ploidies from the biochemical and nutritional perspectives, and we compared the differences between diploid and tetraploid females and males. This is the first report demonstrating differences in nutrient composition between tetraploid *C. gigas* and other ploidies as well as the differences between tetraploid *C. gigas* females and males. The results of this research will provide important information for oyster aquaculture as well as a basis and support for the genetic breeding of oysters.

## 2. Results

### 2.1. Differences in Glycogen, Protein, and Fat Contents among Diploid, Triploid, and Tetraploid Oysters

**Figure 1:**
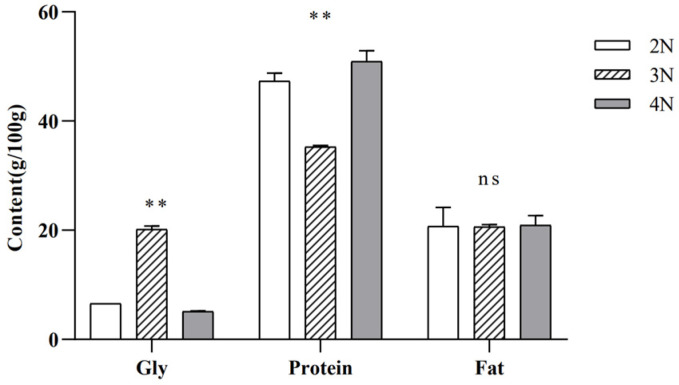
Comparison of protein, glycogen, and lipid contents (g/100 g) between diploid, triploid, and tetraploid *C. gigas*. Note: 2N represents diploids, 3N represents triploids, and 4N represents tetraploids. ** Represents *p* < 0.01, and ns represents undifferentiated.

The contents of the protein and glycogen components of *C. gigas* of various ploidies varied significantly, while the fat content did not show significant differences (Figure 1). The glycogen, protein, and fat contents of diploid *C. gigas* were 6.58 ± 0.01, 47.33 ± 1.46, and 20.74 ± 3.34, respectively. Those of triploids were 20.17 ± 0.59, 35.26 ± 0.28, and 20.65 ± 0.39. The glycogen, protein, and fat contents of tetraploids were 5.11 ± 0.11, 50.94 ± 1.93, and 20.91 ± 1.75, respectively. The glycogen content of triploid *C. gigas* was significantly higher than that of diploids and tetraploids, while glycogen levels in diploids were substantially greater than those in tetraploids. The glycogen content and protein content of different ploidies of *C. gigas* showed opposite trends. Triploid *C. gigas* had considerably lower protein contents than diploids and tetraploids. In comparison to diploids, tetraploids had a much higher protein content.

### 2.2. Differences in Glycogen, Protein, and Fat Contents between Males and Females

**Figure 2:**
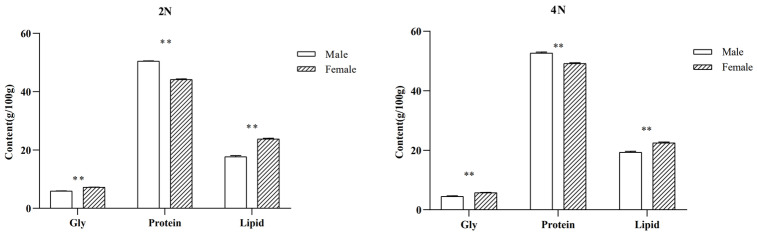
Protein, glycogen, and lipid concentrations in *C. gigas* males and females (g/100 g). Note: 2N represents diploids, 4N represents tetraploids. ** Represents *p* < 0.01.

The biochemical contents of diploid and tetraploid *C. gigas* of different sexes are shown in Figure 2. There were significant differences in glycogen, protein, and fat contents between female and male oysters in both diploids and tetraploids. In diploid oysters, glycogen and lipid contents were significantly lower in males than in females, while protein contents were significantly greater in males than in females. Glycogen content was 5.95 ± 0.02 in males and 7.22 ± 0.03 in females; protein content was 50.49 ± 0.10 in males and 44.17 ± 0.20 in females; and fat content was 17.70 ± 0.39 in males and 23.78 ± 0.24 in females. Similar trends were observed in male and female tetraploid oysters. Male glycogen content (4.50 ± 0.17) was significantly lower than that of female oysters (5.73 ± 0.08); the protein content of males (52.68 ± 0.33) was significantly higher than that of female oysters (49.19 ± 0.21), and lipid content (19.33 ± 0.32) was significantly lower in males than in female oysters (22.49 ± 0.29).

### 2.3. Differences in Amino Acid Contents in C. gigas with Different Ploidies and Sexes

Glutamate was the most prevalent individual amino acid and histidine was the least prevalent in all oyster samples (Table 1). In different ploidies of *C. gigas*, sixteen amino acids were discovered. The total amino acid contents of the five oyster samples ranged from 7.45 to 9.24 g/100 g, and the contents of DAA ranged from 3.48 to 4.22 g/100 g. The EAA contents ranged from 3.03 to 3.92 g/100 g and accounted for 40.64% to 43.12% of the total amino acids. In soft tissues, ploidy comparisons showed that tetraploid and diploid *C. gigas* had significantly higher levels of each amino acid and total amino acid (TAA) content than triploids, except for the amino acids glutamate, alanine, methionine, and proline. Tetraploids had the highest content of EAA and DAA, followed by diploids and triploids, which had the lowest EAA and DAA contents, a result that may be related to gonadal development. Sex-based comparisons showed that in tetraploid and diploid *C. gigas*, females had considerably greater levels of glutamate, methionine, and phenylalanine than males, and they had lower levels of glycine and alanine than males. In addition, female oysters had considerably greater EAA levels than male oysters did (*p* < 0.05; Table 2). The analysis showed that changes in amino acid content were also closely related to reproductive activity.

Amino acid scores (AASs) of oysters of different ploidies and sexes were calculated according to the FAO/WHO amino acid content model, and the findings indicated that all the EAA scores of diploid, triploid, and tetraploid *C. gigas*, except for methionine, were above 100, indicating that the EAA contents of the oysters met the requirements for human consumption and that the protein composition was well-balanced. Methionine is the limiting amino acid of *C. gigas* and does not fulfill human requirements. This is because its content is the lowest among that of all EAAs and is lower than that of the reference amino acid pattern (Table 3 and Table 4). In terms of sex, the amino acid scores of female *C. gigas* were higher than those of males for all EAAs, except lysine.

### 2.4. Differences in Fatty Acid Contents in C. gigas with Different Ploidies and Sexes

The fatty acid composition of oysters of different ploidies is summarized in Table 5. The fatty acid composition of males and females is summarized in Table 6. The fatty acid content of the Pacific oyster were dominated by polyunsaturated fatty acids (PUFAs) and saturated fatty acids (SFAs), each accounting for approximately 40% of the total fatty acids. In all oyster samples, the major SFAs and monounsaturated fatty acids (MUFAs) were oleic acid (C18:0) and palmitic acid (C16:0). As for PUFAs, docosahexaenoic acid (DHA) and eicosapentaenoic acid (EPA) were identified as the major PUFAs. In terms of ploidy, triploid oysters were significantly (*p* < 0.05) higher than diploids and tetraploids in octadecatrienoic acid (ARA), EPA, and ω-3 contents, while triploid and tetraploid oysters were also significantly higher than diploid oysters in DHA content. In terms of sex, female diploid and tetraploid oysters were significantly (*p* < 0.05) higher than males in EPA, DHA, and ω-3 contents, and female oysters also had higher total fatty acid contents. However, the difference between males and females contradicted the findings of Qin et al. (2023) [18]. Tan et al. (2021) demonstrated that the cycle of fatty acid changes in oysters was closely related to the reproductive cycle [19], but there were many differences between populations of the same species. The ratio of ω-3/ω-6 allows for a comparison of the nutritional value of oysters of different ploidies, and a higher ω-3/ω-6 polyunsaturated fatty acid ratio is often used as an indicator of high nutritional value. Both triploid and tetraploid oysters had higher ω-3/ω-6 values than diploids, and both met the FAO experts’ recommendation of dietary ω-3/ω-6 polyunsaturated fatty acid ratios, ranging between 5:1 and 10:1.

**Figure 3:**
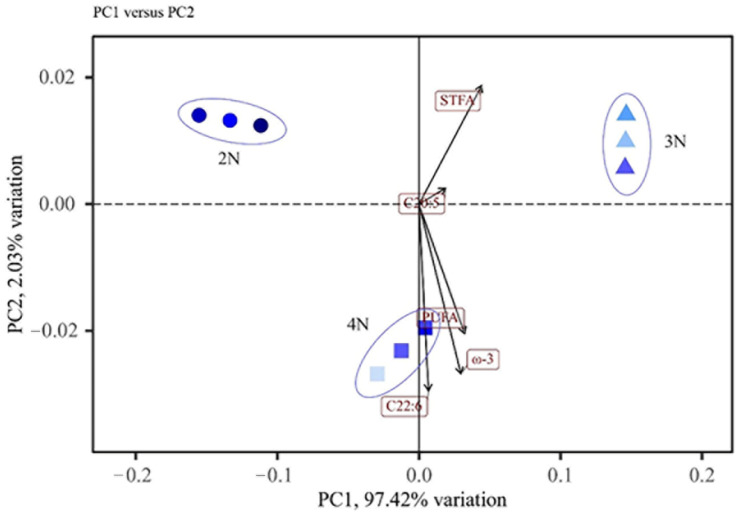
Characterization of amino acid and fatty acid profiles of *C. gigas* of different ploidies by principal component analysis.

The findings unequivocally show that *C. gigas*, particularly triploids and tetraploids, is a good source of critical fatty acids. PCA (Figure 3) was applied to oysters of different ploidies in order to facilitate the interpretation of differences due to oyster ploidy. In Figure 3, diploid, triploid, and tetraploid *C. gigas* are clearly separated, suggesting that most of the differences were due to increases in the genome. This separation was primarily caused by the fatty acid STFA, an ω-3. The findings suggest that diploids, triploids, and tetraploids are important sources of fatty acids.

## 3. Discussion

Triploid oysters have an odd number of chromosome sets, and as such have the characteristic of delayed gametophyte development and reproductive sterility [1], leading to differences in physiological status and nutrient contents between these and other oysters. The reproductive activities of oysters are directly tied to glycogen, one of the significant biochemical components of shellfish, as it is the primary source of energy for gonadal development during reproduction [7]. The accumulation of glycogen is closely related to glycolysis and the TCA cycle [20]. Glycogen can provide energy to oysters quickly and efficiently through hydrolysis, and it serves as the first choice for energy reserves [21]. Glycogen content changes seasonally according to the reproductive cycle of oysters. After spawning is completed, glycogen metabolism is accelerated, and glycogen is gradually accumulated in preparation for the formation and proliferation of gametes [22]. The condition index and gonad weight of oysters decrease significantly during spawning, as demonstrated by Dridi et al., causing the glycogen level of the gonadal reserves to significantly decline; this also demonstrated that the change in glycogen content was strongly associated with gametogenesis and spawning [23]. The findings of this investigation demonstrated that glycogen supplied the necessary energy for gonadal development of *C. gigas*, during which the content sharply decreased. During the reproductive season, triploids, due to their reproductive sterility, consume much less glycogen than diploids. The glycogen content of triploid oysters remains stable during and after the breeding season, allowing the oysters to maintain a fresh, sweet taste year-round and making them more desirable during the breeding season. Protein is an essential component of biosynthesis and enzyme production in all animal tissues [17]. Protein content varied significantly among diploids, triploids, and tetraploids. Protein content in oysters is positively correlated with gonadal development, and protein increases gradually with the formation of gametes [24,25]. This increase in protein content is a result of the large consumption of glycogen and fat in oysters during reproductive activities. The low protein content of triploid oysters may be due to triploids growing substantially more quickly than diploids or tetraploids [1,26], and therefore potentially utilizing the protein stored in the soft body tissues as an energy supply for their growth [27]. Lipids have important effects on the gonadal development of shellfish, as they are the energy substances that maintain gonadal development [28], and thus their storage and utilization are closely related to the reproductive activities of shellfish. The fat content of oysters of different ploidies did not show significant differences. It has been demonstrated that glycogen can be converted into lipids to provide the material requirements for gonadal development [29]. The differences in composition between tetraploid and diploid *C. gigas* may be due to the fact that tetraploid oysters with normal gonadal development carry significantly more eggs than diploid oysters, and the diameter of the eggs is larger than that of diploid oysters. In addition, some of the eggs of tetraploid oysters are dysfunctional [30,31]. The differing energy needs of female and male gametes may account for the disparities between male and female oysters [32]. Glycogen content was significantly different among *C. gigas* of different ploidies, indicating that triploid oysters maintained a high level of glycogen content in their bodies due to low fertility, and also indicating that during gonadal development, a large amount of stored glycogen will be decomposed to provide energy for the process, so that glycogen depletion in the gonadal tissue cells is an inevitable result of gametogenesis. The content of glycogen in the ovary during gametogenesis was significantly higher than that in the spermatheca, a result that was consistent with previous findings [17], indicating that the process of glycogen conversion differed between oyster sexes, and that female *C. gigas* could utilize food better than males to provide energy for reproduction. According to Li et al. (2017), oysters with a high glycogen content could produce more energy and were better able to handle stress. The same oysters also had better flavor, higher quality, and stronger cold tolerance [8]. These differences were also related to summer mortality and energy metabolism [2], suggesting that female oysters were better adapted to changes in the external environment than males and had advantages in energy metabolism, survival, and resilience. The secret to increased larval survival and effective transformation is thought to be fat [33]. In both diploid and tetraploid *C. gigas*, males had significantly lower fat contents than females, also suggesting that female gametes could have a higher survival rate, and also that fat is used as a source of energy during spermatogenesis. This result was also observed previously [34]. Previous research has demonstrated that oysters have the highest fat content in July–August, when the oocytes are fully developed and ready to spawn, suggesting that glycogen may be converted into fat in the oyster ovary [35], possibly allowing female oysters to allocate more proteins to the storage of glycogen and fat. During gametophyte development, proteins are the main structural material in the gonad and mantle region [36], and they also provide nutrients for gamete emission [37]. The results of protein content in various oyster sexes were opposite to those for glycogen and fat contents, indicating that proteins were being accumulated during gonad maturation. Male oysters had a protein level that was substantially higher than female oysters, indicating that gonadal development in male oysters required more energy to sustain and that they also consumed more glycogen and fat.

The analysis showed that changes in amino acid content were also closely related to reproductive activity. However, methionine can only be analyzed in unoxidized hydrolysis products, which are unstable under hydrolysis conditions, resulting in measured values that are lower than the actual methionine content in animals [38]. Therefore, the true methionine score may be greater than 100. It is possible to reasonably mix oysters with methionine-rich diets to achieve amino acid complementarity. The nutritional value of oysters can be fully realized. In addition, amino acids also influence flavor presentation, an important component of the taste of food. In conclusion, diploid, triploid, and tetraploid *C. gigas* are relatively well-balanced in terms of EAA and are good sources of protein. The long-chain ω-3 fatty acid content is necessary for consumers because of its positive health-promoting effects. By lowering cholesterol, it can stop hypertension from occurring [39] and mitigate or prevent the onset of disease [40]. The increase in ω-3 levels in triploid oysters resulted in a lower proportion of SFAs and a higher proportion of PUFAs in triploids than in diploids or tetraploids, suggesting that triploids may provide a greater source of fatty acids for human consumption and thus are healthier for the human body [41]. This study re-emphasizes the importance and usefulness of oyster consumption, as the high fatty acid content is valuable in human nutrition.

## 4. Materials and Methods

### 4.1. Materials and Chemicals

Samples of cultured diploid, triploid, and tetraploid *C. gigas* were obtained from Yantai City, Shandong Province, in August 2022 (during the breeding season). They were all cultured in the same environment of Kongtong Island. Petroleum ether (99%) was obtained from Xilong Science Co., Ltd. (Shantou, China). Sodium citrate (98%), sodium hydroxide (96%), ethanol (98%), sodium chloride (99%), methanol (99%), normal heptane (98%), hydrochloric acid (36%), pyrogallic gallic acid (98%), dichloromethane (99%), and potassium bicarbonate (99%) were obtained from China National Pharmaceutical Group Co., Ltd. (Beijing, China). Anhydrous sodium sulfate (99%), amino acid standard (99%), and fatty acid standard (99%) were obtained from Sigma-Aldrich Trading Ltd. (Shanghai, China).

### 4.2. Sample Preparation

The ploidy of tetraploid, diploid, and triploid oysters was tested by flow cytometry (Beckman Coulter, AD27174, Suzhou, China). Diploid and tetraploid oysters were then sex-determined by carefully opening the shell of the oyster, keeping it as intact as possible to avoid destroying the gonad. A portion of the gamete was scraped from the gonad with a toothpick and gently dotted onto a slide with a drop of seawater. In case the gonads appeared misty or clumped on the slide, they were recorded as male oysters; if they were dispersed in granules, they were recorded as female oysters. The gonads were then reattached to the slide and the sex was reconfirmed using a microscope to identify and avoid hermaphrodites. Approximately 250 g of soft tissue, excluding the adductor muscle, was collected from each group of oysters. The oysters were homogenized and then placed in self-sealing bags in a refrigerator at −80 °C.

Then, 100 g of each of the five oyster samples was freeze-dried (Ningbo Xinzhi Biotechnology Co., Ltd., SCIENTZ-18N, Ningbo, China) for 48 h and then thoroughly grinded into powder form using a mortar and pestle.

### 4.3. Determination of Protein, Glycogen, and Fat Contents

The protein contents of five samples were analyzed using a Kjeldahl nitrogen analyzer (Shanghai Lijing Scientific Instrument Co., Ltd., K9840, Shanghai, China). Approximately 0.4900–0.5000 g of powdered sample was poured into a decoction tube and 5 mL of 98% concentrated sulfuric acid was added. Then, the sample was placed in a preheated graphite digestor (Haineng Future Technology Group Co., Ltd., SH220N, Jinan, China), heated to 360 °C, and then covered with a funnel. The digested sample became white-green after approximately 30 min, followed by another 1 h. Then, 400 g of NaOH was used to prepare 1 L of 10 mol/L NaOH solution. Solution A was prepared by adding bromocresol green (0.1 g) to 100 mL of 95% ethanol. Solution B was prepared by dissolving methyl red (0.2 g) in 100 mL of 95% ethanol. Subsequently, solutions A and B were mixed in a ratio of 3:1. Twenty grams of H_3_BO_4_ was weighed and added to double-distilled water to a volume of 1 L, and 20 mL of methyl red–bromocresol green was added to prepare the indicator. Finally, titration was carried out using 0.1 mol/L sulfuric acid, shaking while titrating, until the solution turned green, and then adding half a drop of sulfuric acid and observing the change in the color of the solution. A grayish-purple color indicated the endpoint of the titration. The protein content was calculated by multiplying the nitrogen content by 6.25.

The glycogen contents of the five groups were analyzed using a commercial kit (Nanjing Jiancheng Institute of Bioengineering, A043-1-1, Nanjing, China) by anthrone colorimetry, with glucose as the standard. Samples weighing 22–23 mg were poured into test tubes, and lye was added separately to obtain a sample-to-lye ratio of 1:3, followed by heating for 0.5 h in a boiling water bath. After that, each sample was diluted to 100 times its initial weight by adding double-distilled water. Subsequently, 100 μL of the hydrolysis product was taken, supplemented with 2 mL of color developer, and heated for 5 min in a boiling water bath. Finally, an enzyme marker (Thermo Fisher Scientific, ZnFINITE200PRO, Reinach, Switzerland) was used to determine the OD value at 620 nm for each sample.

Using Soxhlet extraction to determine crude fat content, 0.2900 g–0.3000 g of sample was weighed into a filter paper tube that was then placed into the extraction tube of the Soxhlet extractor (Jinan Alva Instrument Co., Ltd., SE206, Jinan, China). The receiving bottle was filled with petroleum ether to one-third of the inner volume of the bottle, and the receiving bottle was connected to the extraction tube and heated in a 60 °C water bath. The extraction process lasted for 10 h, during which the titration rate of the petroleum ether was kept at 3 drops/s. After the reaction was terminated, the filter paper cartridge was dried in an oven at 100 °C to a constant weight.

### 4.4. Determination of Fatty Acid and Amino Acid Contents

The determination of amino acids was conducted according to national food safety standard GB5009.124-2016 [42]. A range of 0.9500–1.0000 g of homogenized sample was weighed. Three drops of phenol acid were added along with 10 mL of 6 mol/L hydrochloric. The hydrolyzed tube was then placed in a freezer for 4 min, followed by vacuuming and then filling with nitrogen (this step was repeated three times), and the tube was sealed in the nitrogen-filled state. The sealed hydrolysis tubes were placed in an electric blast-drying oven at 110 °C for 22 h after hydrolysis, and then removed and cooled to room temperature. The hydrolysate was fixed with double-distilled water after being filtered into a 50 mL volumetric flask. A test tube concentrator was used to dry one milliliter of the filtrate at 45 °C while under reduced pressure. The sample was dried, then dissolved in one milliliter of water, dried again under reduced pressure, and lastly evaporated. The test tube was then filled with 2.0 mL of sodium citrate solution (pH 2.2), agitated to dissolve it, and then transferred to the instrument injection bottle for measurement. The sample to be measured and the working solution of the amino acid standard were injected into the amino acid analyzer in the same volume, and the peak areas were used to compute the quantities of amino acids in the sample determination solution.

Fatty acid determination was conducted according to the food national safety standard GB5009.168-2016 [43]. Approximately 1.4900–1.5000 g of the homogenized sample was weighed Then, 2.0 mL of undecylenic triglyceride internal standard solution, 100 mg of pyrogallic gallic acid, 2 mL of 95% ethanol, 4 mL of double-distilled water, and 10 mL of hydrochloric acid 8.3 mol/L were added; the solution was mixed well, and then zeolite was added and the solution was placed in a water bath at 70 °C~80 °C and hydrolyzed for 40 min. The sample was then cooled to room temperature and supplemented with 10 mL of 95% ethanol and mixed well. The hydrolyzed sample was extracted with 50 mL of 1:1 ethyl ether and petroleum ether mixture, and the ether layer extract was collected. This extraction step was repeated three times. The extract was concentrated to dryness using a rotary evaporator, and the remaining material was the fat extract. Sixty milligrams of fat extract was weighed and added to 2.0 mL of internal standard solution. Then, 4 mL of iso-octane was added to dissolve the specimen, and 200 μL of sodium hydroxide methanol solution was added, shaken with vibration for 30 s, and left to stand until clarified. One gram of sodium bisulfate was added to neutralize the potassium hydroxide, and the upper layer of the clear solution in the vial was obtained. The peak area of the chromatogram was used to calculate the amount of fat in the sample solution and fatty acid standard solutions that were put into a gas chromatograph.

### 4.5. Statistical Analysis

Multiple comparisons of biochemical components of different haploid oysters as well as oysters of different sexes were performed using one-way analysis of variance (ANOVA), followed by Duncan’s test using SPSS Statistics 18.0 (SPSS Inc., Chicago, IL, USA). Mean ± standard deviation (M ± SD) was used for all data.

Based on the following formula for the amino acid requirements of preschoolers between the ages of 2 and 5 [44], an amino acid score (AAS) was calculated for each essential amino acid:

$$\mathrm{AAS}=\left(\frac{\mathrm{Test}\;\mathrm{amino}\;\mathrm{acid}}{\mathrm{Reference}\;\mathrm{amino}\;\mathrm{acid}}\right)\times 100$$

## 5. Conclusions

In order to have a comprehensive and deep understanding of the differences among *C. gigas* regarding ploidy and sex, we studied the nutritional composition of diploid, triploid, and tetraploid oysters. The findings indicated that the glycogen content of triploid oysters was obviously greater than that of diploid and tetraploid oysters, and that diploids had a substantially higher glycogen content than tetraploids. Compared to glycogen content, protein content showed opposite results, where tetraploids oysters had the highest protein content, followed by diploids, and triploids had the lowest protein content. In terms of lipids, oysters of different ploidies did not show significant differences. For both diploids and tetraploids, glycogen and lipid contents were significantly lower in males than in females, while protein contents were significantly higher in males. Diploid, triploid, and tetraploid *C. gigas* had essential amino acid scores above 100 and a relatively balanced amino acid composition, except for methionine, indicating that they were a high-protein and high-quality food source. Increased levels of ω-3 PUFAs in triploid oysters resulted in a lower proportion of SFAs and a higher proportion of PUFAs in triploids than in diploids or tetraploids. These results demonstrated that triploid oysters could provide more fatty acids and nutrients for human consumption. Among diploid and tetraploid *C. gigas*, female oysters had higher levels of ω-3 PUFA fatty acids. Males had significantly lower levels of glutamate, methionine, and phenylalanine than females, but higher levels of glycine and alanine than females. In summary, this study is the first report to systematically analyze the differences in fatty acid and amino acid composition among different-ploidy *C. gigas* as well as the difference between diploid and tetraploid male and female oysters. The results provide important information for the application of *C. gigas* in aquaculture as well as a basis for the functional development and genetic breeding of oysters.

## Figures and Tables

**Table 1:** Comparison of amino acid contents (g/100 g) between oysters of different ploidies.

Amino Acid	2N	3N	4N
Aspartic acid ^+^	0.85 ± 0.05 ^a^	0.74 ± 0.01 ^b^	0.88 ± 0.01 ^a^
Threonine *	0.44 ± 0.01 ^a^	0.38 ± 0.01 ^b^	0.47 ± 0.01 ^a^
Serine	0.47 ± 0.01 ^a^	0.36 ± 0.02 ^b^	0.47 ± 0.02 ^a^
Glutamic acid ^+^	1.21 ± 0.01 ^b^	1.15 ± 0.04 ^b^	1.29 ± 0.01 ^a^
Glycine ^+^	0.61 ± 0.01 ^a^	0.49 ± 0.02 ^b^	0.68 ± 0.03 ^a^
Alanine ^+^	0.54 ± 0.01 ^ab^	0.49 ± 0.03 ^b^	0.61 ± 0.02 ^a^
Valine *	0.46 ± 0.00 ^a^	0.40 ± 0.01 ^b^	0.50 ± 0.02 ^a^
Methionine *	0.26 ± 0.01 ^a^	0.23 ± 0.01 ^a^	0.26 ± 0.01 ^a^
Isoleucine *	0.44 ± 0.01 ^a^	0.38 ± 0.01 ^b^	0.46 ± 0.01 ^a^
Leucine *	0.68 ± 0.01 ^a^	0.54 ± 0.00 ^b^	0.70 ± 0.01 ^a^
Tyrosine ^+^	0.34 ± 0.01 ^a^	0.28 ± 0.01 ^b^	0.33 ± 0.01 ^a^
Phenylalanine *^+^	0.42 ± 0.01 ^a^	0.34 ± 0.00 ^b^	0.42 ± 0.01 ^a^
Lysine *	0.74 ± 0.02 ^a^	0.56 ± 0.01 ^b^	0.78 ± 0.02 ^a^
Histidine *	0.23 ± 0.00 ^a^	0.21 ± 0.01 ^b^	0.24 ± 0.01 ^a^
Arginine	0.61 ± 0.04 ^a^	0.44 ± 0.02 ^b^	0.65 ± 0.04 ^a^
Proline	0.44 ± 0.01 ^b^	0.50 ± 0.01 ^a^	0.49 ± 0.00 ^a^
TAA	8.67 ± 0.11 ^b^	7.45 ± 0.01 ^c^	9.16 ± 0.01 ^a^
EAA	3.64 ± 0.02 ^b^	3.03 ± 0.06 ^c^	3.79 ± 0.03 ^a^
DAA	3.95 ± 0.02 ^b^	3.48 ± 0.01 ^c^	4.19 ± 0.00 ^a^

* Essential amino acid. + Delicious amino acid. Different letters in the same row indicate statistical differences between samples at the *p* < 0.05 level.

**Table 2:** Comparison of amino acid contents (g/100 g) between oysters of different sexes.

Amino Acid	2N		4N	
	Male	Female	Male	Female
Aspartic acid ^+^	0.74 ± 0.04 ^a^	0.95 ± 0.06 ^a^	0.82 ± 0.01 ^b^	0.95 ± 0.01 ^a^
Threonine *	0.40 ± 0.01 ^b^	0.48 ± 0.01 ^a^	0.46 ± 0.01 ^a^	0.49 ± 0.01 ^a^
Serine	0.40 ± 0.01 ^b^	0.54 ± 0.01 ^a^	0.44 ± 0.01 ^a^	0.49 ± 0.03 ^a^
Glutamic acid ^+^	1.08 ± 0.01 ^b^	1.35 ± 0.03 ^a^	1.21 ± 0.01 ^b^	1.38 ± 0.04 ^a^
Glycine ^+^	0.71 ± 0.04 ^a^	0.51 ± 0.01 ^b^	0.79 ± 0.04 ^a^	0.57 ± 0.02 ^b^
Alanine ^+^	0.58 ± 0.01 ^a^	0.50 ± 0.01 ^b^	0.67 ± 0.02 ^a^	0.54 ± 0.02 ^b^
Valine *	0.42 ± 0.00 ^b^	0.49 ± 0.00 ^a^	0.49 ± 0.02 ^a^	0.50 ± 0.01 ^a^
Methionine *	0.22 ± 0.01 ^b^	0.29 ± 0.01 ^a^	0.22 ± 0.01 ^b^	0.28 ± 0.00 ^a^
Isoleucine *	0.40 ± 0.01 ^b^	0.48 ± 0.01 ^a^	0.45 ± 0.02 ^a^	0.48 ± 0.01 ^a^
Leucine *	0.62 ± 0.01 ^b^	0.72 ± 0.03 ^a^	0.68 ± 0.01 ^a^	0.72 ± 0.01 ^a^
Tyrosine ^+^	0.30 ± 0.01 ^b^	0.38 ± 0.01 ^a^	0.31 ± 0.01 ^a^	0.35 ± 0.01 ^a^
Phenylalanine *^+^	0.36 ± 0.01 ^b^	0.47 ± 0.00 ^a^	0.38 ± 0.00 ^b^	0.45 ± 0.01 ^a^
Lysine *	0.72 ± 0.01 ^a^	0.75 ± 0.03 ^a^	0.79 ± 0.04 ^a^	0.75 ± 0.00 ^a^
Histidine *	0.22 ± 0.01 ^b^	0.24 ± 0.00 ^a^	0.23 ± 0.00 ^a^	0.24 ± 0.01 ^a^
Arginine	0.62 ± 0.03 ^a^	0.58 ± 0.04 ^a^	0.70 ± 0.04 ^a^	0.59 ± 0.02 ^a^
Proline	0.42 ± 0.01 ^a^	0.46 ± 0.02 ^a^	0.47 ± 0.00 ^a^	0.51 ± 0.00 ^a^
TAA	8.17 ± 0.00 ^b^	9.16 ± 0.22 ^a^	9.08 ± 0.02 ^a^	9.24 ± 0.06 ^a^
EAA	3.35 ± 0.02 ^b^	3.92 ± 0.06 ^a^	3.69 ± 0.04 ^b^	3.88 ± 0.02 ^a^
DAA	3.75 ± 0.04 ^a^	4.14 ± 0.08 ^a^	4.16 ± 0.04 ^a^	4.22 ± 0.04 ^a^

* Essential amino acid. + Delicious amino acid. Different letters in the same row indicate statistical differences between samples at the *p* < 0.05 level.

**Table 3:** Essential amino acid scores of oysters of different ploidies.

Amino Acid	2N	3N	4N
Threonine	126.87	127.52	128.28
Valine	106.11	94.67	109.17
Methionine	85.68	88.21	81.10
Isoleucine	126.87	127.52	125.55
Leucine	112.04	103.55	109.17
Phenylalanine + Tyrosine	146.10	138.70	136.46
Lysine	155.19	136.67	154.82

**Table 4:** Essential amino acid scores of oysters of different sexes.

Amino Acid	2N		4N	
	Male	Female	Male	Female
Threonine	122.40	131.00	126.65	132.58
Valine	102.82	106.99	107.93	108.23
Methionine	76.94	90.46	69.23	86.58
Isoleucine	122.40	131.00	123.90	129.87
Leucine	108.41	112.29	106.99	111.32
Phenylalanine + Tyrosine	134.64	154.66	126.65	144.30
Lysine	160.23	148.87	158.19	147.58

**Table 5:** Comparison of fatty acid content (g/100 g) between oysters of different ploidies.

Fatty Acid	2N	3N	4N
C14:0	0.117 ± 0.00 ^b^	0.110 ± 0.00 ^c^	0.123 ± 0.00 ^a^
C16:0	0.534 ± 0.00 ^a^	0.510 ± 0.00 ^b^	0.512 ± 0.00 ^b^
C16:1	0.130 ± 0.00 ^a^	0.107 ± 0.00 ^c^	0.114 ± 0.00 ^b^
C17:0	0.024 ± 0.00 ^c^	0.030 ± 0.00 ^a^	0.025 ± 0.00 ^b^
C18:0	0.079 ± 0.00 ^c^	0.090 ± 0.00 ^a^	0.083 ± 0.00 ^b^
C18:1	0.211 ± 0.01 ^c^	0.290 ± 0.00 ^a^	0.232 ± 0.00 ^b^
C18:2	0.045 ± 0.00 ^b^	0.050 ± 0.00 ^a^	0.046 ± 0.00 ^b^
C18:3	0.076 ± 0.00 ^c^	0.090 ± 0.00 ^a^	0.083 ± 0.01 ^b^
C20:4 (ARA)	0.025 ± 0.00 ^b^	0.030 ± 0.00 ^a^	0.026 ± 0.00 ^b^
C20:5 (EPA)	0.296 ± 0.01 ^c^	0.370 ± 0.00 ^a^	0.327 ± 0.00 ^b^
C22:6 (DHA)	0.262 ± 0.00 ^b^	0.290 ± 0.00 ^a^	0.289 ± 0.00 ^a^
SFA	0.759 ± 0.01 ^a^	0.750 ± 0.00 ^a^	0.755 ± 0.01 ^a^
MUFA	0.325 ± 0.00 ^b^	0.357 ± 0.00 ^a^	0.326 ± 0.00 ^b^
PUFA	0.705 ± 0.01 ^c^	0.832 ± 0.00 ^a^	0.770 ± 0.01 ^b^
Σω-3	0.635 ± 0.01 ^c^	0.752 ± 0.00 ^a^	0.699 ± 0.01 ^b^
Σω-6	0.070 ± 0.00 ^b^	0.081 ± 0.00 ^a^	0.071 ± 0.00 ^b^
STFA	1.804 ± 0.02 ^c^	1.98 ± 0.00 ^a^	1.870 ± 0.02 ^b^

Different letters in the same row indicate statistical differences between samples at the *p* < 0.05 level.

**Table 6:** Comparison of fatty acid content (g/100 g) between oysters of different sexes.

Fatty Acid	2N		4N	
	Male	Female	Male	Female
C14:0	0.05 ± 0.01 ^b^	0.19 ± 0.00 ^a^	0.06 ± 0.00 ^b^	0.19 ± 0.01 ^a^
C16:0	0.31 ± 0.01 ^b^	0.76 ± 0.00 ^a^	0.36 ± 0.00 ^b^	0.66 ± 0.01 ^a^
C17:0	0.02 ± 0.00 ^b^	0.03 ± 0.00 ^a^	0.02 ± 0.00 ^a^	0.03 ± 0.00 ^a^
C18:0	0.05 ± 0.01 ^b^	0.10 ± 0.00 ^a^	0.07 ± 0.01 ^b^	0.10 ± 0.00 ^a^
C16:1	0.04 ± 0.01 ^b^	0.22 ± 0.00 ^a^	0.06 ± 0.00 ^b^	0.17 ± 0.00 ^a^
C18:1	0.13 ± 0.00 ^b^	0.29 ± 0.00 ^a^	0.18 ± 0.00 ^b^	0.28 ± 0.01 ^a^
C18:2	0.03 ± 0.00 ^b^	0.06 ± 0.00 ^a^	0.03 ± 0.00 ^b^	0.06 ± 0.00 ^a^
C18:3	0.04 ± 0.00 ^b^	0.11 ± 0.00 ^a^	0.06 ± 0.00 ^b^	0.11 ± 0.00 ^a^
C20:4 (ARA)	0.02 ± 0.00 ^b^	0.03 ± 0.00 ^a^	0.02 ± 0.00 ^a^	0.03 ± 0.00 ^a^
C20:5 (EPA)	0.19 ± 0.01 ^b^	0.40 ± 0.00 ^a^	0.25 ± 0.00 ^b^	0.40 ± 0.01 ^a^
C22:6 (DHA)	0.22 ± 0.01 ^b^	0.31 ± 0.00 ^a^	0.26 ± 0.00 ^b^	0.31 ± 0.01 ^a^
SFA	0.42 ± 0.01 ^b^	1.10 ± 0.01 ^a^	0.52 ± 0.01 ^b^	0.99 ± 0.02 ^a^
MUFA	0.16 ± 0.00 ^b^	0.49 ± 0.00 ^a^	0.22 ± 0.00 ^b^	0.43 ± 0.01 ^a^
PUFA	0.49 ± 0.02 ^b^	0.92 ± 0.01 ^a^	0.63 ± 0.01 ^b^	0.91 ± 0.03 ^a^
Σω-3	0.45 ± 0.02 ^b^	0.82 ± 0.00 ^a^	0.58 ± 0.01 ^b^	0.82 ± 0.03 ^a^
Σω-6	0.05 ± 0.00 ^b^	0.09 ± 0.00 ^a^	0.05 ± 0.01 ^b^	0.09 ± 0.00 ^a^
STFA	1.09 ± 0.04 ^b^	2.52 ± 0.01 ^a^	1.39 ± 0.02 ^b^	2.35 ± 0.06 ^a^

Different letters in the same row indicate statistical differences between samples at the *p* < 0.05 level.

## Data Availability

Data are contained within the article.
